# Physicochemical and hydration properties of different cereal and legume gluten‐free powders

**DOI:** 10.1002/fsn3.1170

**Published:** 2019-08-20

**Authors:** Nohed Boucheham, Laurence Galet, Séverine Patry, Mohammed Nasreddin Zidoune

**Affiliations:** ^1^ Laboratoire de Nutrition et Technologie Alimentaire (LNTA), Institut de la Nutrition, de l’Alimentation et des Technologies Agro‐Alimentaires (INATAA) Université des Frères Mentouri Constantine 1 Constantine Algeria; ^2^ Laboratoire RAPSODEE UMR CNRS 5302, IMT Mines Albi Université de Toulouse Albi France

**Keywords:** gluten‐free powders, physicochemical properties, sorption isotherms, thermodynamic properties, water diffusion, water holding

## Abstract

**Background:**

The wetting and hydration stage is the key step in manufacture process of several cereal‐based products. Knowledge of hydration properties of gluten‐free ingredients can contribute to improve the quality of gluten‐free products. The objective of the present work was to investigate hydration properties for a large variety of gluten‐free ingredients. Powders of tow gluten‐free cereals (rice and maize) and powders of tow legumes (chickpea and faba bean) in comparison with durum wheat semolina. The hydration properties were evaluated at 25°C by vapor and liquid water addition.

**Results:**

Legume powders had the highest sorption capacity and stronger interaction with vapor water. Rice showed the highest vapor water diffusion at all RH intervals. Water holding capacity, swelling kinetics, and immersion enthalpy in liquid water were higher for legume and maize powders.

**Conclusion:**

Gluten‐free cereal powders show hydration properties different from those of legumes. Different combinations of these gluten‐free materials can be made to approach the properties of wheat powders.

## 1 INTRODUCTION

Celiac disease is now considered a relatively common disease affecting about 0.6%–1% of the world's population (Makharia et al., 2014). For celiac disease patients, adhering to a restrictive gluten‐free diet can be challenging for several reasons. First, food choices are essentially limited because cereal products are staple foods and play a predominant role in a regular diet. Second, a wide range of processed foods contain gluten‐based products as additional ingredients (Missbach et al., 2015). Hence, a requirement to produce high‐quality and a wide variety of gluten‐free products is as important as ever. For that reason, there has been a growing interest in the search of ingredients for the production of gluten‐free cereal‐based products (Fiorda, Soares Júnior, Da Silva, Souto, & Grosmann, 2013; Padalino, Conte, & Del Nobile, 2016). Gluten replacement constitutes a technological challenge, since commercial gluten‐free products exhibit quality deficiencies such as low volume, poor color, an undesirable texture, and lower nutritional value compared with their wheat counterparts (Matos & Rosell, 2011; O’Shea, Arendt, & Gallagher, 2014).

Rice and maize are the preferred ingredient sources for making gluten‐free foods due to their abundance, low cost, and their suitability for celiacs (Arendt, O’Brien, Schober, Gormley, & Gallagher, 2002; Gimenez et al., 2013). However, they are limited in terms of their nutritional properties (Matos & Rosell, 2011). Moreover, they have relatively poor technological properties for interaction and development of a cohesive network. In this sense, the purpose of adding natural rich‐protein ingredients such as legume flours is mainly for improving nutritional quality and maintaining a strong cohesive structure (Arendt et al., 2002; Ribotta, Ausar, & Morcillo, 2004). Legumes are, after cereals, the most cultivated plants in the world and represent an important source of gluten‐free proteins (Berrios, Morales, Cámara, and Sánchez‐Mata (2010). They are low in fat, high in resistant starch content, and excellent sources of dietary fiber and micronutrients, such as iron, zinc, potassium, and folate. The combination of cereal and legume proteins would provide better overall essential amino acid balance and good nutritional value (Eggum & Beame, 1983).

In order to obtain good quality from alternative materials, it is often necessary to balance formulations and adequate technological production processes have to be adopted to counteract any changes in the rheological properties caused by substitution of gluten (Arendt et al., 2002). It has been well documented that the wetting and hydration step is the critical factor in many manufacturing processes of cereal‐based products such as pasta, couscous, and bread (Barkouti, Delalonde, Rondet, & Ruiz, 2014; Iveson, Litster, Hapgood, & Ennis, 2001; Oulahna, Hebrard, Cuq, Abecassis, & Fages, 2012). During hydration, the solid–water interactions lead to binding between particles for the formation of agglomerates and dough (Hébrard et al., 2003; Roman‐Gutierrez, Guilbert, & Cuq, 2002; Saad et al., 2009). In the literature, it has also been reported that substances that swell in water could replace gluten in the dough (Sivaramakrishnan, Senge, & Chattopadhyay, 2004). Given this importance, the hydration properties of gluten‐free ingredients compared with commercial durum wheat semolina constitute an important field of research. Sorption isotherms, water diffusion coefficients, and liquid–solid immersion are some techniques that permit the characterization of solid hydration properties and understanding of hydration mechanisms. The knowledge of thermodynamic functions could provide information on the affinity between water and the powders including the binding forces, the water molecules, their spatial arrangement, and the spontaneity of the sorption process (Hébrard et al., 2003; Murrieta‐Pazos, Galet, Patry, Gaiani, & Scher, 2014; Oulahna et al., 2012). The objective of this study was therefore to contribute to better understanding the mechanisms of hydration of some natural gluten‐free powders by vapor or liquid water addition. For this, we chose to study two gluten‐free cereal powders (rice and maize) and tow legume powders (chickpea and faba bean) of physical and chemicals properties which can be used in different formulations to improve both nutritional and technological aspects.

## 2 MATERIALS AND METHODS

### 2.1 Materials

Superior quality industrial semolina and native gluten‐free materials: two cereals, polished rice and yellow maize and two legumes, chickpea and dehulled faba bean, were purchased from local supermarkets in Constantine, Algeria. The grains of rice, maize, chickpea, and beans were grinded using a mill grinder (MG E3, UMA Rouiba—Algeria, type MG E3, 1410 rpm) and passed through a sieve of 0.8 mm mesh size. Al samples were stored in hermetically sealed containers at 4°C until use. Measurements of initial content of water, ash, and lipids content were determined according to the French standards NF V03‐707, NF V03‐720, 03‐760, respectively (AFNOR Recueil de norms, 1991). The protein content was calculated after multiplication by conversion factor (5.7), the total nitrogen determined according to the Kjeldahl method (NF V03‐750; Barr et al., 1995). Starch contents were measured by the polarimetry method.

### 2.2 Physical determinations

Particle size distribution of samples was measured using laser granulometry (Mastersizer 2000 Malvern Instruments) at room temperature. Powders are characterized by volume‐equivalent diameters for which the number of particles is inferior to 10(d10), 50 (d50), and 90% (d90) of the total number of particles. The span is calculated as (d90‐d10)/d50.

Apparent and tapped density of samples is determined in triplicate using a nitrogen pycnometer (Ultrapycnometer 1000, Quantachrome Instruments) for apparent density.

Color measurement was carried out in duplicate on semolina and gluten‐free powders using a chromameter (Minolta CR‐450; Minolta Corp). Values for L* (lightness on a scale of 100 for pure white to 0 for black), a* (red), and b* (yellow) were recorded, and mean values were reported.

### 2.3 Scanning electron microscopy

The powders were observed with a Field‐Emission Environmental Scanning Electron Microscope (XL30; FEI/Philips) operating at 20 kV. Powders were spread onto a double‐sided adhesive carbon disk fixed on a support. Samples were directly observed with a secondary electron detector.

### 2.4 Hydration properties by vapor water

#### 2.4.1 Dynamic vapor sorption

Sorption isotherms of powders were obtained gravimetrically using an automated Dynamic Vapor Sorption system (DVS 1000‐Surface Measurement Systems, UK) equipped with a controlled atmosphere microbalance. The experiments were carried out at constant temperature (25°C) with different RH values ranging between 0% and 95%. Approximately 50 mg of powder was loaded onto the quartz sample pan. After a pre‐equilibration at ERH = 0% by continuous flow of dry air, the samples were submitted to a 10‐step hydration process at successive levels of relative humidity (from10% to 95%). The samples were considered to be at equilibrium when the value dm/dt (slope of the changing in mass with time) was set to be0.005 mg/min or equilibration time exceeded 300 min. Monolayer values as well as multilayer thermodynamic properties were calculated by different sorption models and then compared. Experimental data were mathematically modeled with the following sorption isotherm models: GAB (Guggenheim–Anderson–de Boer), Y&N (Young &Nelson), Freundlich, and Smith. The isotherm equations for models used to fit the data are presented in Table 1 (Al‐Muhtaseb, McMinn, & Magee, 2004; Isa, Lang, Asaari, Aziz, & Ramli, 2007; Murrieta‐Pazos et al., 2014). The quality of fit was evaluated by correlation coefficient (*R* ^2^).

**Table 1:** Isotherm equations for experimental data fitting

Model	Mathematical expression
GAB	*X* = *X*_0_ *Ka_w_*/[(1−*Ka_w_*)(1−*Ka_w_*+*CKa_w_*)]
Y&*N*	$M_{S}=A\left(\theta +\alpha \right)+B\varphi$
	$M_{D}=A\left(\theta +\alpha \right)+B\theta {\text{RH}}_{\max}$
	$A=\frac{\rho_{w}V_{\text{ads}}}{D}$
	$B=\frac{\rho_{w}V_{\text{abs}}}{D}$
	$\theta =\frac{\text{RH}}{\text{RH}+\left(1-\text{RH}\right)E}$
	$\alpha =-\frac{\text{ERH}}{E-\left(E-1\right)\text{RH}}+\frac{E^{2}}{\left(E-1\right)}\ln\frac{E-\left(E-1\right)\text{RH}}{E}-\left(E+1\right)\ln\left(1-\text{RH}\right)$
	φ=θRH
	$E=e^{-\frac{q_{1}-q_{l}}{k_{g}T}}$
Freundlich	X* = *KP*^1/n^*
Smith	X = A + (B log(1— *a*_w_))

Note

For GAB equation; *X* is the moisture content, *X*_0_ is the monolayer moisture content, C and K the GAB model constants. For Y&*N*: *M*_S_ and *M*_D_ are equilibrium moisture contents for the respective cycle at each relative humidity, and RH_max_ is the maximum exposed relative humidity, ρ_w_ is the density of water at the experimental temperature; *D* is the sample dry weight, and *V*_ads_ and *V*_abs_ the volumes of adsorbed and absorbed water, q_1_ is the heat of adsorption of water bound to the surface of the sample, q_L_ is the heat of condensation of water molecules, k_B_ is Boltzmann's constant, and T is the absolute temperature. For Smith: X is the moisture content, A the quantity of water in the first sorbed fraction, and B the quantity of water in the multilayer moisture fraction. For Freundlich: K is the Freundlich capacity factor, 1/*n* is the Freundlich intensity parameter, p: Equilibrium pressure of adsorbate

#### 2.4.2 Water diffusion coefficient

The basic equation to describe the transport of small solvent molecules in a polymer is Fick's second law of diffusion (Equation 1) which can be used to calculate the diffusion coefficient of water vapor into different powder samples (Equation 2) using the same experimental protocol of the DVS previously described (Murrieta‐Pazos et al., 2014; Oulahna et al., 2012).

(1) $$F=-D_{f}\cdot \frac{\delta c}{\delta x}$$

(2) $$\frac{M_{t}}{M_{\text{eq}}}=1-\frac{6}{\pi^{2}}\sum_{i}w\left(a_{i}\right)\sum_{n=1}^{\infty }\frac{1}{n^{2}}\exp\left(\frac{-D\cdot n^{2}\cdot \pi^{2}\cdot t}{a_{i}^{2}}\right)$$

The apparent diffusion coefficient of vapor water into powders is obtained at a given relative humidity (X%) by plotting ln (*M*_eq_—*M*_t_) as a function of time t. The diffusion coefficient at a given relative humidity (X%) is calculated from the slope of the linear part of this curve.

(3) $$\ln\left(M_{\text{eq}}-M_{t}\right)=\ln\left(\frac{8M_{\text{eq}}}{\pi }\right)-\frac{\pi^{2}\cdot D_{f}}{e^{2}}\cdot t$$

where *M*_t_ is the water content (kg of water/kg dm) at time *t* (min), *M*_eq_ is the water content (kg of water/kg dm) at equilibrium (*t *= ∞), *a*_i_ is the radius (m) of the particle i; *w*(*a*_i_) is the weight fraction of particles that are characterized by the radius; *n* is the calculation increment, and *e* is the thickness of film of powder.

### 2.5 Hydration properties by liquid water

#### 2.5.1 Water holding capacity (WHC)

Powders samples (1 g) were placed in centrifuge tubes, and 10 ml distilled water were added. After stabilization for 30 min at 25°C with continuous stirring, suspensions were centrifuged (3,000 g; 20 min). The supernatants were dried at 105°C. WHC was calculated according to the following equations (Doporto, Dini, Mugridge, & Vina, 2012; Tananuwong & Malila, 2011). The measurements were conducted in triplicate.

(4) $$\text{WHC}\left(g/\text{gdb}\right)=\frac{\left(\text{weight of precipitate paste}\left(g\right)-\text{Sample weight}\left(g\right)\right)\times 100}{\text{Sample weight}\left(\text{gdb}\right)\times \left(100-\text{solubility}\right)}$$

(5) $$\%\text{Solubility}=\frac{\text{weight of dried supernatant}\left(g\right)\times 100}{\text{Sample weight}\left(\text{gdb}\right)}$$

#### 2.5.2 Kinetics of swelling

A sample of 20 g of each powder is poured into a 100‐ml graduated cylinder containing 50 ml of distilled water at 25°C. The test tube is capped, and 10 successive turns are made so as to hydrate all the particles. 50ml of water is added to lower the particles remained stuck along the wall. It is left to rest at 25°C, and then, the volume is noted after 5, 10, 20, 30, 40, 50, and 60 min. Swelling index (SI) was calculated according to the following equations (Guezlane & Abecassis, 1991). The measurements were conducted in triplicate.

(6) $$\text{SI}=100\times \left(V_{f}-V_{i}\right)/V_{i}$$

where *V*_i_ is volume of sample (ml) at 0 min. And *V*_f_ is volume of sample (ml) at different times.

#### 2.5.3 Thermodynamic hydration properties by mixing calorimetry

The hydration energy under mixing conditions by immersion of a powder in water is measured by mixing microcalorimetry technique. Hydration energy of powder by liquid or vapor water could be assimilated to the energy required to bring into contact water and grain surface and to homogenize the mixture. A sample of 100 mg, exactly weighed, were mixed with 1,000 mg of distilled water using a C80 D microcalorimeter (Setaram).Immersion enthalpy (Δ*H*_imm_) of powders in water were measured at 25°C. The measurement was repeated 5 times for each powder (Oulahna et al., 2012).

### 2.6 Statistical analysis

Data were expressed as mean ± *SE*. Statistical analysis was performed using Excel software. One‐way analysis of variance (ANOVA) and Tukey's test were used to determine significant differences at *p* < .05 for multiple samples by means of XLSTAT (Microsoft).

## 3 RESULTS AND DISCUSSION

### 3.1 Biochemical, structural, and physicochemical composition

The biochemical properties of semolina and gluten‐free powders rice, maize, chickpea, and faba bean are reported in Table 2. The selected powders offer a large diversity in biochemical composition. An initial moisture content of semolina (16.41%) is clearly higher than gluten‐free powders (14.78%, 8.51%, 12.22%, and 11.75% for rice, maize, chickpea, and faba bean, respectively). At this level, it cannot be said that the durum wheat semolina is more hygroscopic because the milling process to obtain the industrial semolina is different from the process that we used to obtain gluten‐free powders. Also, the methods of obtaining different dry grains of rice, maize, chickpea, and faba bean are different.

**Table 2:** Biochemical composition of the semolina and gluten‐free powders (contents in g/ 100 g dry base)

	Water	Starch	Proteins	Lipids	Ash
Semolina	16.41 ± 0.15	68.88 ± 0.30	12.14 ± 0.19	0.84 ± 0.13	0.85 ± 0.03
Rice powder	14.78 ± 0.41	87.97 ± 0.39	8.58 ± 0.73	0.34 ± 0.17	0.30 ± 0.06
Maize powder	8.51 ± 0.85	61.43 ± 0.51	11.90 ± 0.23	4.52 ± 0.14	2.23 ± 0.17
Chickpea powder	12.22 ± 0.02	39.09 ± 0.59	23.97 ± 0.62	5.91 ± 0.91	3.36 ± 0.55
Faba bean powder	11.75 ± 0.51	32.86 ± 0.64	29.05 ± 1.20	1.89 ± 0.39	3.12 ± 0.41

Concerning the components that must contribute to strengthen interactions with water (starch, proteins, ash), cereal powders (durum wheat semolina, rice, and maize) are characterized by higher contents in starch (61.43%–87.97%) than legumes (32.86%–39.09%). For the protein and ash contents, the cereal powders are characterized by lower values (8.58%–12.14% and 0.30%–2.23%, respectively) in comparison with legumes (23.97%–29.05% and 3.12%–3.36%). The chickpea and maize powders are characterized by relatively high values of lipids content which are hydrophobic component. Similar compositions have been reported by the literature (Amir, Haenni, & Youyou, 2007; Koehler & Wieser, 2013; Petitot, Boyer, Minier, & Micard, 2010).

Physical properties of semolina and gluten‐free powders are presented in Table 3. Different particle size distributions were found, and cereal powders (rice and maize) are characterized by high D_50_ values ranging from 200 to 260 µm and low size dispersion ranging from 2.54 to 2.77 in comparison to the legume powders with D_50_ ranging from 82 to 170 µm and spam ranging from 7.82 to 4.09. It can be noticed that the legumes studied are more friable than cereals. High particle size was measured for durum wheat semolina (D_50_ = 506 µm) in comparison with published values (204–300 µm) (Hébrard et al., 2003; Landillon, Cassan, & Morel, 2008).

**Table 3:** Particle size distribution, apparent and true particle density, and color parameters (a*, b*, L*)

	Particle size		Density (g/cm^3^)		Color		
	D_50 (µm)_	Span (D_90_‐D_10_/D_50_)	Tapped density	Apparent density	a*	b*	L*
Semolina	506	1.22	0.78^b^ ± 0.01	1.44^b,c^ ± 0.0001	49.43^a^ ± 0.04	50.48^a^ ± 0.21	91.20^b^ ± 0.07
Rice powder	268	2.54	0.82^a^ ± 0.02	1.46^a^ ± 0.0002	51.22^a^ ± 1.65	26.95^d^ ± 0.16	105.22^a^ ± 3.66
Maize powder	200	2.77	0.55^e^ ± 0.01	1.40^c^ ± 0.0016	49.38^a^ ± 0.37	49.89^a^ ± 0.62	90.49^b^ ± 0.62
Chickpea powder	82	7.32	0.71^c^ ± 0.01	1.44^a,b^ ± 0.0002	51.50^a^ ± 0.07	48.29^b^ ± 0.40	89.66^b^ ± 0.03
Faba bean powder	170	4.09	0.66^d^ ± 0.01	1.42^b,c^ ± 0.0002	49.63^a^ ± 0.01	38.00^c^ ± 0.05	95.08^b^ ± 0.04

Note

Means with same letter within column are not significantly different (*p* < 0.05).

Concerning density, significantly different tapped density values (*p* ˂ .05) were found for all powders, and they vary between 0.55 and 0.82 g/cm^3^ (tab.3). Both legume powders which have the lower tapped density values in comparison with cereals represent an interparticle space more than 50% (50.69% and 53.52% for chickpea and faba bean, respectively) while it is less than 50% for semolina (45.83%) and rice powder (43.84%). The bulk density depends on the attractive interparticle forces, particle size, and number of contact positions (Peleg & Bagley, 1983). The apparent density values did not differ significantly for all samples except rice which had highest starch content and lower protein content. This might have been due to the fact that protein has lower density than starch granule (Kuakpetoon, Flores, & Milliken, 2001). Maize powder is characterized by lower density (0.55 g/cm^3^ and 1.40 g/cm^3^ for tapped and apparent density, respectively) and high interparticle space (60%).

Color of semolina‐based foods is an important quality parameter for consumer acceptability. A bright yellow color is commonly preferred. Color values of powder samples are presented in Table 3. No significant difference (*p *˂ .05) was observed for a* and L* values except for the rice powder which has the highest lightness index due to its white color. However, different values of the yellow index (b*) were observed for the different samples. Durum wheat semolina and maize powder have the highest values of b* (50.48 and 49.89, respectively) followed by chickpea (48.29), then faba bean (38.00), and finally rice powder (26.95). Different yellowness indexes of different powders are due to the different contents of xanthophylls (carotenoids), the pigments causing this natural coloring.

**Figure 1:**
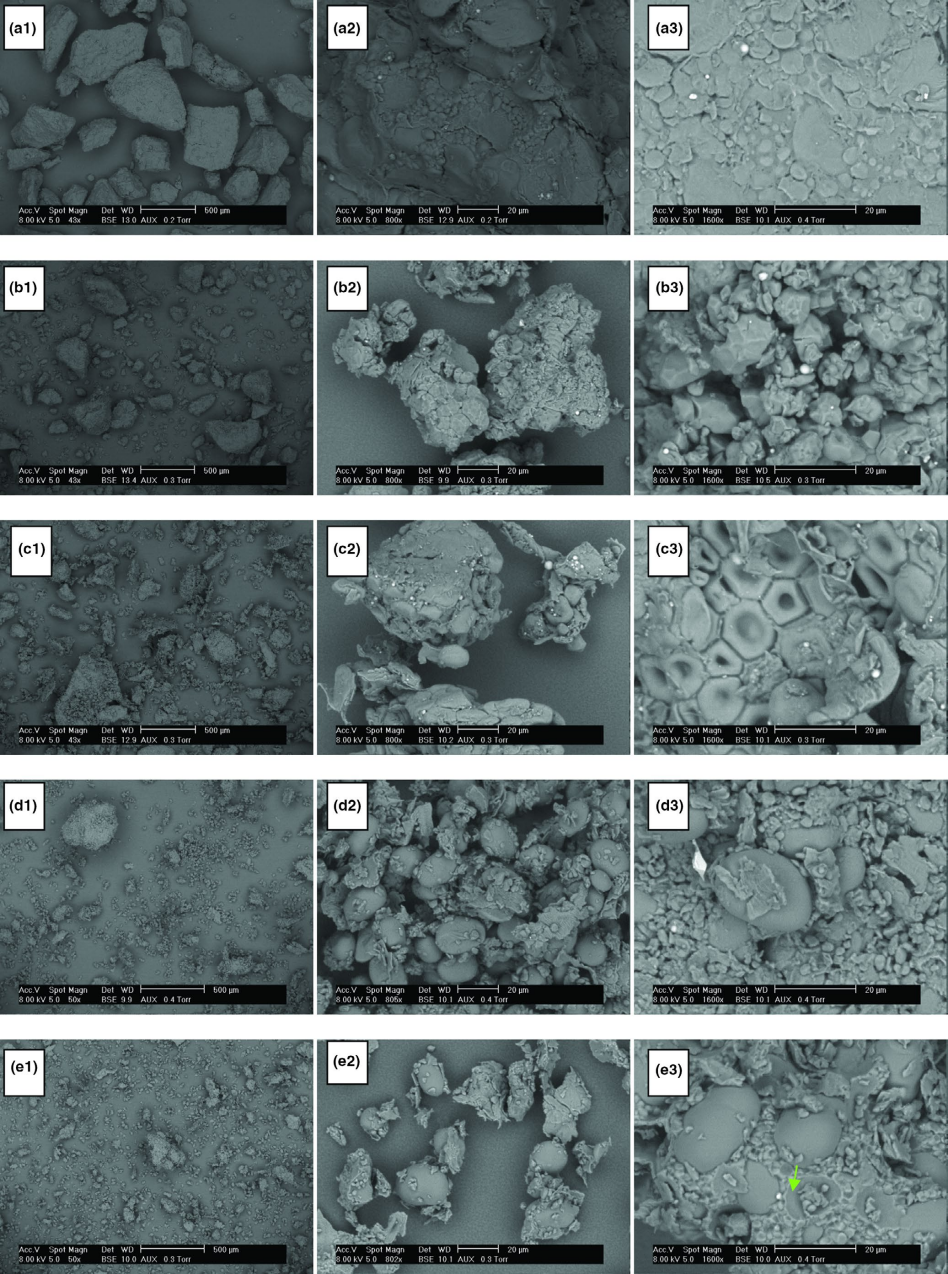
Characterization of Microstructure of native powders particles of semolina (a), rice (b), maize (c), chickpea (d), and faba bean (e) particles by *SEM* (scanning electron microscopy)

SEM micrographs of native particles of the different raw materials are presented in Figure 1. From these images, it clearly appears that semolina is characterized by smooth surfaces without cracks and starch granules are embedded in a protein network. While, surfaces are rough for gluten‐free powders and pores can be observed specially in the case of rice powder (b3). Besides, several starch granules of gluten‐free powders are isolated without being damaged, which reflects a weak cohesion between the starch and the protein network. The size and shape of the particles is very heterogeneous, and the particles of durum wheat semolina have a size much higher than that of other gluten‐free powders.

With regard to morphology, the semolina starch is characterized by the presence of two populations of granules: the smallest (5–10 μm) spherical and large (25–40 μm) lenticular (photo a_3_). The rice starch is composed of polyhedral starch granules of size between 3 and 9 μm (b_3_). Maize (c_3_) is composed of polygonal shape starch granules with angles, concave in the center and of size varying between 3 and 12μm. Both legumes are composed of egg‐shaped starch granules with a size of 10 to 22μm for chickpeas (d_3_) and 10 to 50μm for beans (e_3_) (Buléon, Colonna, Planchot, & Ball, 1998; Juliano, 1993).

### 3.2 Sorption isotherms

**Figure 2:**
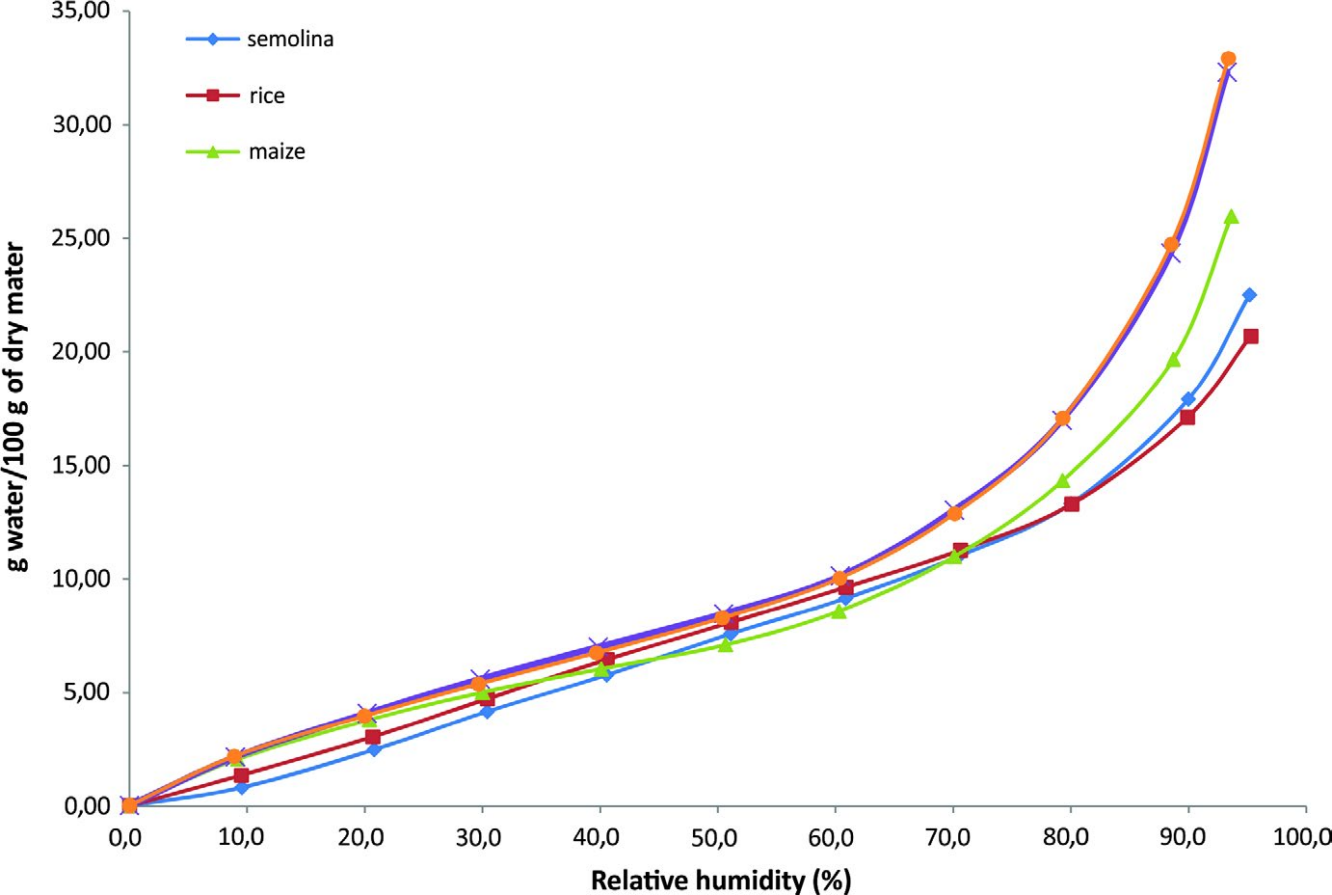
Adsorption isotherms of semolina and gluten‐free powders at 25°C

The adsorption isotherm profiles at 25°C, from 0% to 95% RH are presented in Figure 2 for the gluten‐free food powders (rice, maize, faba bean, and chickpea) compared with semolina. As expected, these isotherms demonstrate an increase in equilibrium moisture content (EMC) with increasing relative humidity for the five powders. Similar curves shapes for semolina,(Erbas, Ertugay, & Certe, 2005; Hébrard et al., 2003; Murrieta‐Pazos et al., 2014) rice,(Bingol, Prakash, & Pan, 2012; Toğrul & Arslan, 2006) maize,(Oyelade, Tunde‐Akintunde, Igbekac, Okeb, & Rajid, 2008; Samapundo et al., 2007) and legume seeds or legume flowers(Menkov, 2000; Moreira, Chenlo, Torres, & Prieto, 2010; Nikolay & Menkov, 2000) have been reported in the literature. The two legume powders show the same behavior and adsorbing the same amounts of water for each relative humidity range. However for cereals, semolina and rice curves were parallel between 0% and 80%, nevertheless, rice values are slightly higher than semolina. Beyond 80%, water uptake of rice decreases from semolina. For maize, we noticed 3 different areas, the first between 0% and 30% where maize curve is almost superimposed on those of legumes. From 30 to 70% of HR, maize behavior is close to the other cereals (semolina and rice). Then, the maize water uptake increases at an intermediate level between legumes at the top and cereals (rice and semolina) at the bottom for higher values of RH.

According to the characteristics of the different components present in the solids, different regions can be identified depending on the expected liquid/solid interactions in a specific range of RH. The initial convex region (region I) is the monolayer region in which the water is bound by hydrogen bonds on the polar sites of the solids. In this region, water is most strongly adsorbed and is immobile. The monolayer is formed in the ERH range of 0% to 20% for the five food powders. Region II corresponds to the linear portion of the isotherm. Here, the water forms several additional layers and corresponds to water held by the components. In the last concave region (region III), condensed water is held in weakly bound states, is mobile, and is classically designed as bulk phase water.

Sorption capacity, Figure 2 shows that legume powders, faba bean, and chickpea have higher water adsorption capacities than cereal powders (32.92 g/100g dm chickpea and 32.31 g/100g dm for faba bean against 21.02 et 20.33 g/100g dm, 25.96 g/100g dm for semolina, rice and maize, respectively) all through relative humidity range (0%–95%). This may be due to the higher ash content in legume powders compared with cereals and especially to the high protein content. The affinity of a biological substance for water is a function of the number of polar groups and their accessibilities in the molecules. The water adsorption sites in the carbohydrate molecules are essentially OH hydroxyls. For proteins, two different types of polar sites can be considered, the polar sites of the amino acid side chains, each of which can bind up to 6 molecules of water and the polypeptide backbone which is the major adsorption zone. Overall, in a complex system, the behavior of each type of compound vis‐à‐vis the water affects the behavior of the whole mixture. For the difference in particle size, Saad et al. (2009) observed only a very small variation in the adsorption capacity of the regrind fractions of durum wheat flour. The classification of samples according to the sorption capacity at a relative humidity greater than 70% is as follows: Chickpea ≈ faba bean ˃ maize ˃ semolina ≈ rice.

**Figure 3:**
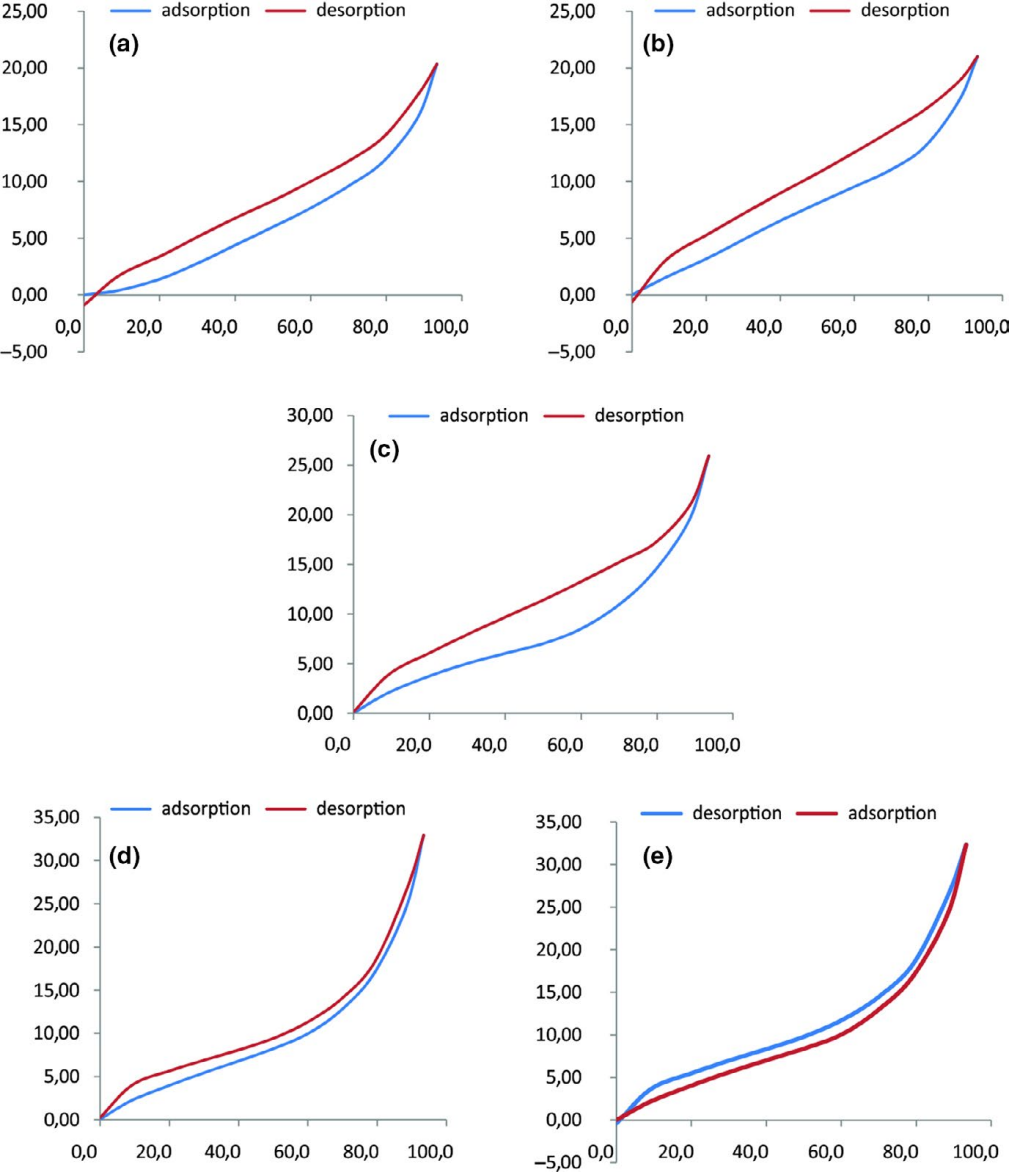
Hysteresis effect for semolina (a), rice (b), maize (c), chickpea (d), and faba bean (e)

The hysteresis phenomenon is shown for the five powders (Figure 3) to a varying degree but with the similar shape. The hysteresis loops (Figure 3) extended over the total relative humidity range and are closed between the upper and lower limits of RH. This is in agreement with a typical hysteresis shape observed for organic, nonrigid solids such as pharmaceutical and food materials. The desorption data were higher than the adsorption data, and the moisture sorption hysteresis effect was more significant for cereals than legumes. The magnitude of hysteresis is related to the nature and state of the components of food (Andrade, Lemus, & Pérez, 2011), reflecting their potential for structural and conformational rearrangements which alters the accessibility of energetically favorable polar sites (Andrade et al., 2011). Therefore, vapor adsorption induces more structural and conformational changes in cereals than legume powders. Maize is the most reactive. The hysteresis effects can also be attributed to the bulk absorption accompanied by swelling effects.

### 3.3 Water vapor sorption mathematical modeling

Several mathematical models exist to describe water sorption isotherms of food materials; no one equation gives accurate results throughout the entire range of water activities, and for all types of foods (Al‐Muhtaseb, McMinn, & Magee, 2002). Four sorption isotherm models (GAB, Y&N, Freundlich, and Smith) were used to analyze interactions between the water vapor and the five food powders: semolina, rice, maize, faba bean, and chickpea. The parameters of the equations and correlation coefficient *R* ^2^ are given in Table 4. All the selected models gave suitable fits (*R* ^2^ ranging from 0.935 to 0.999) apart GAB equation with rice powder. Although, it has been reported that the GAB model is the best to predict food isotherm (Al‐Muhtaseb et al., 2002; Andrade et al., 2011; Timmermann & Chirife, 1991); however, it does not happen for all (Erbas et al., 2005). They noted that fitness of GAB at 20°C was not good for semolina and farina. The three parameters‐Y&N model gave the best fit for the five food powders (*R* ^2^ ≥ 0.99). The goodness of fit of a sorption model to experimental data does not describe the nature of the sorption process, and it only reflects on the mathematical quality of the model (Samapundo et al., 2007). Also as water is associated with the food matrix by different mechanisms in different a_w_ regions, no single model can be considered accurate over the entire a_w_ range.

**Table 4:** Fitting parameters for mathematical models applied to sorption data of semolina and gluten‐free powders

Model	Parameters	Semolina	Rice	Maize	Chickpea	Faba bean
GAB	X_m_(kg/kg dm)	0.08	0.18	0.05	0.06	0.06
	C	2.78	2.75	8.29	6.98	6.36
	K	0.65	0.44	0.85	0.90	0.84
	R^2^	0.998	0.807	0.955	0.984	0.958
Y&*N*	A (kg/kg dm)	0.03	0.03	0.03	0.05	0.04
	E	0.58	0.33	0.48	0.99	0.99
	B (kg/kg dm)	0.07	0.08	0.10	0.01	0.02
	R^2^	0.998	0.999	0.991	0.990	0.991
Freundlich	B1 (kg/kg dm)	0.22	0.20	0.26	0.34	0.33
	B2	0.59	0.73	0.52	0.45	0.47
	R^2^	0.973	0.966	0.935	0.938	0.936
Smith	B1 (kg/kg dm)	0.08	0.07	0.09	0.11	0.11
	B2 (kg/kg dm)	0.01	0.02	0.01	0.01	0.01
	R^2^	0.991	0.980	0.993	0.991	0.991

Using the GAB equation, it is possible to calculate monolayer moisture content *X*_m_ and adsorption constants C and K, which are related to the energies of interaction between the first and the further sorbed molecules of water at the individual sorption sites. *X*_m_ values of semolina and rice powder (0.08 and 0.18 kg/kg dm, respectively) are higher than those of maize, faba bean, and chickpea powders (0.05–0.06 kg/kg dm). This may be owed to the fact that maize, faba bean, and chickpea have higher fat contents than semolina and rice. The presence of fat at the surface of particles powder decreases the number of hydrophilic sites able to adsorb water molecules. Indeed, several authors observed a negative correlation between the fat content and water vapor adsorption (Bianco, Boente, Pollio, & Resnik, 2001; Roca, Guillard, Guilbert, & Gontard, 2006). Also, rice powder has *X*_m_ value markedly higher and this may be due to the high content of starch. The starch has the higher GAB *X*_m_ value compared with the other components of wheat flour such as pentosans and gluten (Roman‐Gutierrez et al., 2002). The monolayer of the five powders were ranged between 0.05 and 0.18 kg/kg dm, in generally for starchy foods, the *X*_m_ of GAB values that vary from 0.03 to 0.16kg/kg dm and for legume seeds 0.044–0.086 kg/kg dm (Aguerre, Viollaz, & Suarez, 1996; Furmaniak, Terzyk, & Gauden, 2007; Moreira et al., 2010). Constant C of GAB refers to the interaction of water with surface. As expected (Table 4), maize, faba bean, and chickpea powders with higher values of C (8.26, 6.36 and 6.98, respectively) have a greater binding energy than semolina and rice (2.78 and 2.75) although they have less sorption sites available (lower values of *X*_m_). Constant K of GAB falls into the range of 0.44–0.90 and shows the same trend as the parameter “C,” that is, higher for maize, faba bean, and chickpea powders. Similar values of C and/or K of GAB have already been reported in the literature for semolina (Erbas et al., 2005; Murrieta‐Pazos et al., 2014), for rice (Toğrul & Arslan, 2006) for maize, (Quirijns, Boxtel, Loon, & Straten, 2005) and for legumes (Ayranci & Duman, 2005; Rahman, Perera, & Thebaud, 1998). Finally, if GAB constants calculate 0 ˂ K ˂ 1 and C ˃ 2, sorption isotherms are type II (Blahovec, 2004).

The Y&N model showed the best correlation with all powders (*R* ^2^ ≥ 0.99). A_Y&N_ is equivalent to *X*_m_ in the GAB equation and represent monolayer moisture capacity, B_Y&N_ is related to the amount of moisture absorbed by the sample, and E_Y&N_ is an energy term relating to the strength of water vapor interaction to the surface of the sample and is similar to C of GAB (Murrieta‐Pazos et al., 2014). We can observe that the monolayer values for the five powders were close (0.03–0.05 kg/kg dm) and lower than those of GAB. A_Y&N_ for both legumes faba bean and chickpea were slightly higher than those of three cereal powders. However, B_Y&_ *_N_* values of tree cereal powders (0.07, 0.08, and 0.10 kg/kg dm for semolina, rice, and maize, respectively) were clearly higher than dose of legume powders (0.02 for faba bean and 0.01 for chickpea). This indicated that cereal powders absorb more water than legumes. The Y&N model considered a monolayer with a lesser quantity of water molecules, and the rest of the humidity is taken into account in the B_Y&N_ parameter as absorbed water which has not similar term in the GAB equation (Murrieta‐Pazos et al., 2014). This may explain the difference between the results obtained by the two models. As with GAB, E_Y&N_ indicates a stronger interaction between water vapor molecules and the surface of legume powders than cereals.

In Smith equation, B2_smith_is the quantity of water in the first fraction of sorbed water which exhibits a higher than normal heat of condensation and B1_smith_ is the quantity of water in the second fraction which consists of multilayer of condensed water molecules (Al‐Muhtaseb et al., 2002; Andrade et al., 2011). B2_smith_ value for rice is slightly greater than that determined for the other food powders. This is consistent with the results of the *X*_m_ of GAB where rice presented the highest value. However, all B2_smith_ values are clearly lower than the values of *X*_m_ of GAB and A_Y&N_. The amount of condensed water in multilayer fraction B1_smith_ was higher for legumes (0.11 kg/kg dm) than three cereals (0.07 kg/kg dm for rice, 0.08 for semolina, and 0.09 for maize). We note that this classification follows the same powders ranking order of their sorption capacity in the third region of isotherms which corresponds to bulk water.

The Freundlich model is not limited to the formation of a complete monolayer. This equation describes heterogeneous systems and reversible adsorption. If Freundlich intensity parameter B2_Freundlich_˂1, the adsorption is favorable and the adsorption capacity increases and new adsorption sites appear (Isa et al., 2007). B2_Freundlich_ values are less than 1 for semolina, rice, maize, faba bean, and chickpea.

### 3.4 Moisture diffusivity

**Figure 4:**
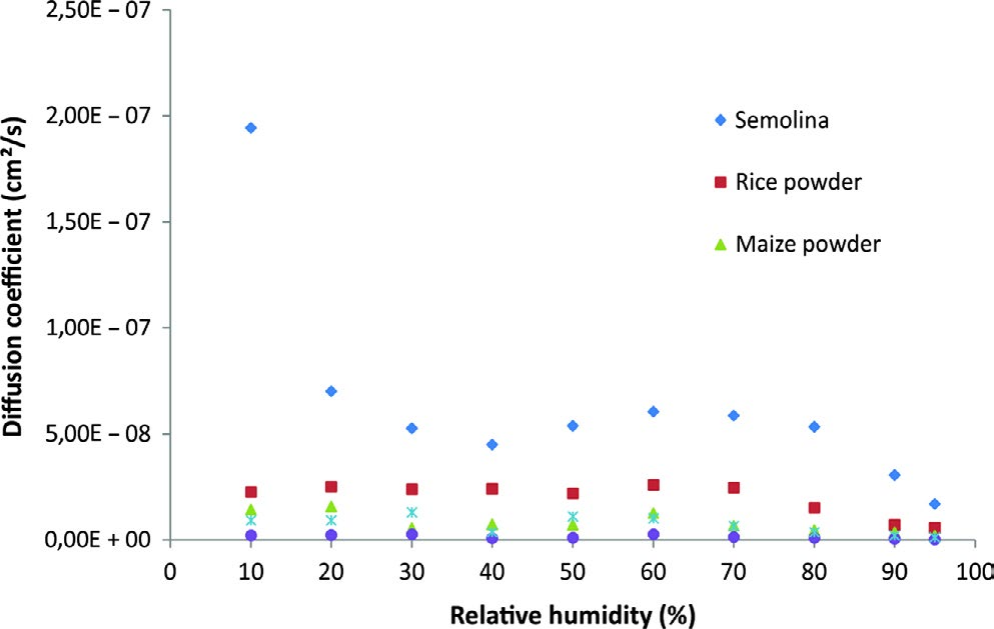
Variation of the diffusion coefficients at 25°C in semolina and gluten‐free powders

Food products are multiphase and heterogeneous materials. Mass transfer in these systems is often interpreted as a diffusive phenomenon (Peppas & Brannon‐Peppas, 1994). The evolution of the diffusion coefficients versus relative humidity of semolina and gluten‐free powders is shown in Figure 4. Similar curves were obtained for starch‐based materials durum wheat semolina (Chivrac, Angellier‐Coussy, Guillard, Pollet, & Avérous, 2010; Oulahna et al., 2012). For the HR lower than 30%, the diffusion coefficient decreases, and then, it increases for the interval 30%–70% of HR and finally another decrease to a minimum for RH 95% is found. For gluten‐free powders, the diffusion started slowly until 10% RH and slightly varied below 70% RH. These behaviors were found for small particles (0–315 µm) of semolina, and a good correlation of particle size with water diffusion was found (Murrieta‐Pazos et al., 2014). Indeed, at 90% RH, semolina composed with large particles (D_50_ = 506µm) had the highest diffusion coefficient 2.77 × 10^–8^ cm^2^/s compared with 0.96 × 10^–8^, 0.24 × 10^–8^, 0.14 × 10^–8^, and 0.04 × 10^‐8^cm^2^/s for rice (D_50_ = 268µm), corn (D_50_ = 200µm), chickpea (D_50_ = 170µm), and faba bean (81.9µm), respectively. On the other hand, water diffusion in maize powder was lower than rice powder, although the particles of the two powders have a similar size. This can be explained by difference in lipid content, and the moisture diffusivity of sponge cake decreases with increasing lipid content (Roca et al., 2006). Contrary to results which show that the starch and the damaged starch have the lowest diffusion coefficients (0.67 × 10^–15^ m^2^/s and 0.37 × 10^–15^ m^2^/s, respectively) compared with gluten (1.52 × 10^–9^cm^2^/s),( Roman‐Gutierrez et al., 2002) our semolina and rice powder with the highest levels of starch have the highest water diffusion coefficients. Finally, maize, chickpea, and faba bean powders which have the lowest water diffusion coefficients presented the strongest water–solid interaction. Therefore, the high affinity for water should slow down its diffusion in the solid.

### 3.5 Water holding capacity (WHC)

**Figure 5:**
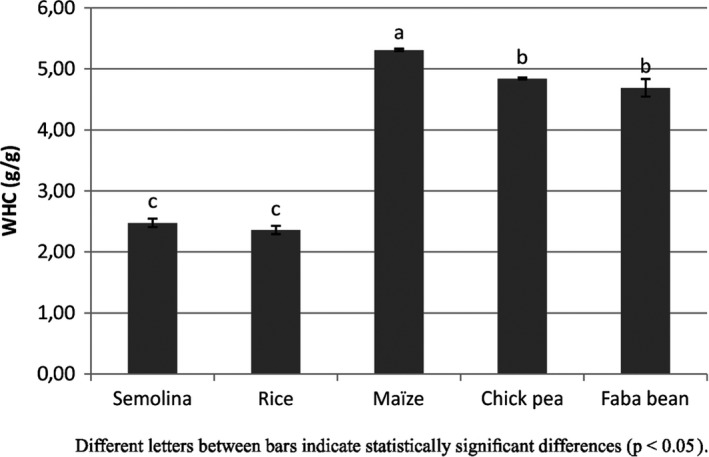
Holding capacity (g water/ g sample) of semolina and gluten‐free powders

Water absorption capacity of flours indicates how some nutrients and bioactive compounds interact with water. The pentosans, which are very hydrophilic, can absorbs 15 times their weight in liquid water, while, the gluten absorbs 2,15 times its weight, the native starch 0.44 times its weight and damaged starch 2 times its weight in liquid water for wheat flour (Feillet, 2000). Maize powder WHC (5.31 ± 0.02g water/g sample) at 25°C was significantly (*p* ˂ .05) higher than the values found for the other two cereals powders, durum wheat semolina (2.48 ± 0.07 g/g), and rice powder (2.36 ± 0.07 g/g) (Figure 5). So, this observation could be due to the higher dietary fiber content in maize powder that may effectively contribute to increasing water holding capacity. Maize meal contains 12.19% total dietary fiber compared with 2.4% and 2.3% for durum wheat semolina and rice, respectively (Juliano, 1993; Petitot et al., 2010). Both legumes powders have WHC higher than semolina and rice but significantly lower than maize (4.84 ± 0.02 g/g for chickpea and 4.69 ± 0.14 g/g for faba bean). These results are consistent with those reported for soy flour (4.79–6.75 g of water/g of dry matter; Heywood, Myers, Bailey, & Johnson, 2002). These results are in agreement with those of vapor sorption except for maize, which absorbs more liquid water than water vapor.

### 3.6 Kinetics of swelling

**Figure 6:**
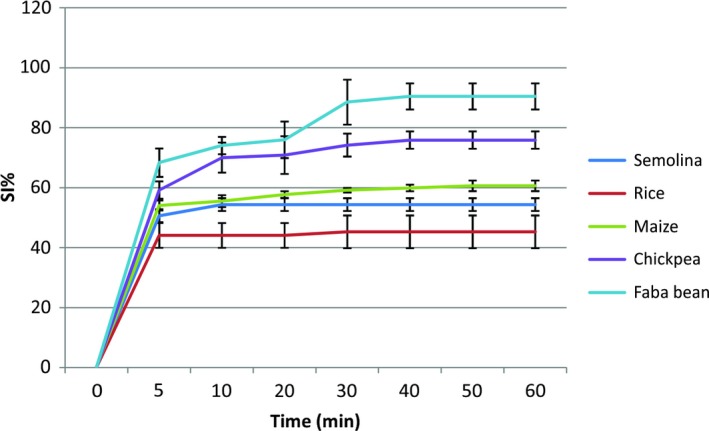
Kinetics of swelling at 25°C for semolina, rice powder, maize powder, chickpea powder, and faba bean powder

The results of swelling index showed that both legumes (90.39 ± 4.34% for faba bean and 75.83 ± 2.89% for chickpea) swell more than cereals (60.60 ± 1.68% for maize, 54.32 ± 2.14% for semolina, and 45.24% ± for rice powder) (Figure 6). The ranking obtained, chickpea and faba bean ˃ maize ˃ semolina ˃ rice is very well matched the results of water vapor sorption at RH = 95%.

The evolution of the swelling index as a function of time at 25°C for the different powders studied shows three stages: at the first step, the SI increases quickly during the first 5 min; this is due to rapid absorption of water by the upper layers of the particles of the different powders. The second stage lasts longer, and the increase in the SI is less important, especially for cereals: This may be due to the packing effect of the powders and the slower diffusion of the water in the middle of the particles. We note that semolina SI stabilizes rapidly after 10 min followed by rice powder after 20 min, while chickpea and faba bean continue to swell up to 40 min and corn until the 50th minute. This is in good agreement with the diffusion coefficient, which is much higher for semolina and rice than for the other three powders.

### 3.7 Thermodynamic hydration properties by mixing calorimetry

Immersion enthalpy (ΔH_imm_) of durum wheat semolina, rice, maize, chickpea, and faba bean powders in water determined by mixing microcalorimetry at 25°C is given in Table 5.

**Table 5:** Immersion enthalpy at 25°C

	Semolina	Rice	Maize	Chickpea	Faba bean
ΔH_imm_ (J/g db) à 25°C	−2.04^a^ ± 0.18	−6.46^b^ ± 0.90	−13.96^c^ ± 4.28	−6.20^ab^ ± 0.69	−3.65^ab^ ± 1.31

Note

Means with same letter within column are not significantly different (*p* < 0.05).

Immersion in water of all studied powders is an exothermic reaction (ΔH_imm_ ˂ 0). Maize powder presents the higherΔH_imm_, about −13.96 J/g. Immersion of semolina and faba bean powder in water gives the lowest amounts of heat (−3.65 J/g and −2.04 J/g db, respectively). All the enthalpies obtained for our five powders are integrated in the interval constituted by the two major components of semolina, gluten (proteins) with a ΔH_imm_ = −2 J/g and starch ΔH_imm_ = −17.2 J/g. Immersion enthalpy is not influenced by the size of the grains, the quality of semolina, the temperature, or the nature of the liquid of hydration. It essentially depends on biochemical composition, and it is correlated with wetting energy, bringing into contact water molecules and solid surface (Oulahna et al., 2012). Gluten presents a higher immersion enthalpy (wetting contact angle (θ): starch/water (θ = 38°) ˂ gluten/water (θ = 85°)), its components having better wetting properties. Therefore, we can say that maize powder has the best wetting properties followed by rice and chickpea and finally faba bean and durum wheat semolina (Oulahna et al., 2012). For as biochemical composition, we noticed that the immersion enthalpy is not affected by the starch content; the rice which contains the largest amount of starch (87.69% db) has a comparable ΔH_imm_ with that of chickpea and faba bean which have low levels of starch (39.51% and 32.40% db, respectively). And finally, we note that semolina, which had the highest coefficient of diffusion, had the lowest enthalpy of immersion.

## 4 CONCLUSION

The results show distinct characteristics for the selected gluten‐free powders. Firstly, both gluten‐free cereals, rice and maize, have very different physicochemical and hydration properties. The rice powder has a higher density and a higher lightness but a very low yellow index compared with the maize powder. But maize powder adsorbs more water vapor, absorbs more liquid water, and swells more than rice. The thermodynamic parameters calculated by the different mathematical models of the sorption isotherms or by immersion calorimetry show that the interaction forces between the maize powder and the vapor, or liquid water are greater than the interaction between water and the rice powder. In general, it is the rice which has the characteristics closest to that of durum wheat semolina. The powders of both legumes have comparable physicochemical and hydration properties except for the yellow index, which is high for chickpea compared with faba bean. Both powders show greater sorption and interaction with water vapor than semolina, rice, and maize. With liquid water, chickpea, and faba bean absorb and swell more than cereals, but the interaction forces are comparable with those of semolina and rice. In conclusion, good diversity is observed between the physicochemical and hydration properties of the studied gluten‐free powders. This offers a great choice in the construction of different gluten‐free formulas that come closest to the properties of durum wheat semolina in order to obtain end products of good quality.

## CONFLICT OF INTEREST

The authors declare that they do not have any conflict of interest.

## ETHICAL APPROVAL

This study does not involve any human or animal testing.
